# NCAPD2 is a favorable predictor of prognostic and immunotherapeutic biomarker for multiple cancer types including lung cancer

**DOI:** 10.1186/s41021-023-00291-4

**Published:** 2024-01-03

**Authors:** Linyuan Feng, Yang Yang, Zhenhua Lin, Minghua Cui, Aihua Jin, Aili Cui

**Affiliations:** 1 Yanbian University Hospital, Yanji, China; 2 Key Laboratory of Pathobiology of High Frequency Oncology in Ethnic Minority Areas, Yanbian University, State Ethnic Affairs Commission, Yanji, China

**Keywords:** NCAPD2, Prognosis, Immune infiltration, Tumor microenvironment, Bioinformatics

## Abstract

**Background:**

Non-SMC condensin I complex subunit D2 (NCAPD2) belongs to the chromosomal structural maintenance family. While the different contribution of NCAPD2 to chromosome in mitosis have been thoroughly investigated, much less is known about the expression of NCAPD2 in pan-cancer. Thus, we used a bioinformatics dataset to conduct a pan-cancer analysis of NCAPD2 to determine its regulatory role in tumors.

**Methods:**

Multiple online databases were analyzed NCAPD2 gene expression, protein level, patient survival and functional enrichment in pan-cancer. Genetic alteration and tumor stemness of NCAPD2 were analyzed using cBioPortal and SangerBox. The GSCA and CellMiner were used to explore the relationship between NCAPD2 and drug sensitivity. The diagnostic value of prognosis was evaluated by ROC curve. Subsequently, the immune infiltration level and immune subtype of NCAPD2 in lung adenocarcinoma (LUAD) and lung squamous cell carcinoma (LUSC) were analyzed using TIMER1 and TISIDB.

**Results:**

NCAPD2 gene expression was significantly higher in most cancers and associated with clinical stage and poor prognosis. Genomic heterogeneity of NCAPD2 promoted the occurrence and development of tumors. GO enrichment analysis suggested NCAPD2 might be involved in DNA repair and immune response. NCAPD2 was involved in immune infiltration of LUAD and LUSC. ROC curves showed that NCAPD2 has important prognosis diagnostic value in LUAD and LUSC. Moreover, NCAPD2 was drug sensitive to topotecan, which may be an optimize immunotherapy.

**Conclusions:**

It was found that NCAPD2 was overexpressed in pan-cancers, which was associated with poor outcomes. Importantly, NCAPD2 could be a diagnostic marker and an immune related biomarker for LUAD and LUSC.

**Supplementary Information:**

The online version contains supplementary material available at 10.1186/s41021-023-00291-4.

## Introduction

Tumorigenesis is a complex process involving oncogene activation, genomic heterogeneity and epigenetic modification [1]. Genomic instability contributes to tumor evolution by heterogeneity under endogenous or exogenous selection pressures [2]. Tumor heterogeneity is mainly reflected in epigenetics, which is involved in regulating gene expression and promoting tumor development [3]. Nonetheless, the concept of tumor heterogeneity also applies to the diverse microenvironments in which tumor cells interact [4]. This microenvironment is critical for tumor initiation or growth. Therefore, immunotherapy approaches with less toxicity and better responses are of immediate need in the future.

The tumor microenvironment (TME) is a complex tissue microenvironment which is composed of tumor infiltrating immune cells (TIICs), stromal cells, other secretory molecules and extracellular matrix [5]. Tumor infiltrating lymphocytes (TILs) mainly play a role in immune monitoring and kill cancer cells in TME, and the activation of T cells in TILs can finely tune excessive damage and autoimmunity responses [6, 7]. In the meantime, dendritic cells (DCs) can regulate immune response and cancer-associated fibroblasts (CAFs) can increase the proliferation of tumor cells [8, 9]. Recently, cancer immunotherapy can effectively treat various cancers, but it does not work well for the majority of patients among certain cancer types [10, 11]. Thus, it is urgent need to detect a biomarker to assess the response to these immunotherapies.

Some researchers found that there are three subunits of NCAPD2, NCAPG and NCAPG on chromosome 12p13.3, which together form the condensing protein complex I. During the cell cycle, the involvement of NCAPD2 in mitotic chromosome condensation and segregation has been primarily studied as one of its key functions [12, 13]. In the past few years, some articles reported that NCAPD2 appears to act a part in neurodegenerative disorders, such as Alzheimer’s, autism and Parkinson’s disease [14]. In HCC, NCAPD2 not only acted as a hub gene, but also had diagnostic value in tumor tissues [15]. Jing et al. found that NCAPD2 inhibited autophagy and promoted the proliferation and migration of colorectal cancer cells *via* regulating Ca^2+^/CAMKK/AMPK/mTORC1 pathway and PARP-1/SIRT1 axis [16]. In non-small-cell lung cancer, NCAPG has been identified as a prognostic biomarker of immune infiltration [17]. Whereas, the relationship between NCAPD2 and immune infiltration remains unclear. These researches might be useful to detect the role of NCAPD2 for immunotherapy in pan-cancer especially lung cancer.

**Fig. 1:**
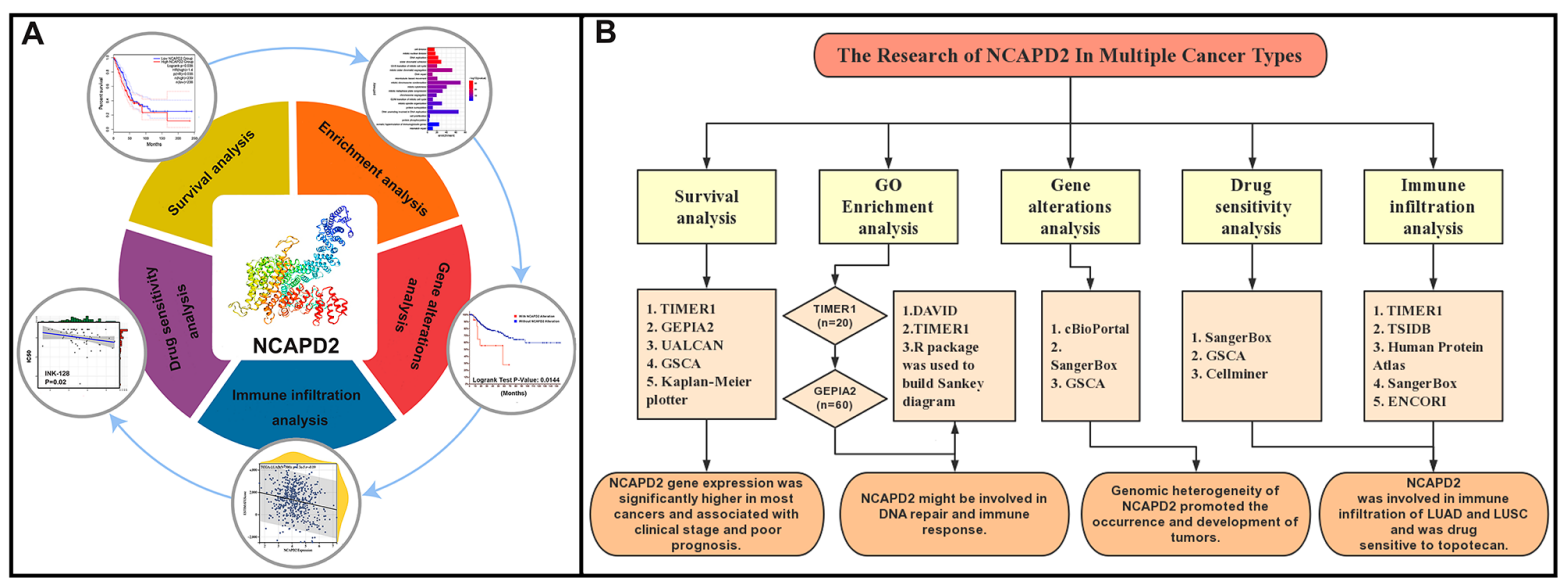
Summary of the Pan-cancer Analysis of NCAPD2

Here, we investigate the expression of NCAPD2 in a variety of cancers and its impact on the survival of patients with different cancers, and analyze the biological processes it may be involved in. To further investigate the expression levels of the NCAPD2 gene when it is mutated in different cancers, and then explore its sensitivity to different anticancer drugs. Finally, its potential application as a predictor of pan-cancer immunotherapy response was analyzed to establish new possibilities (Fig. 1).

## Materials and methods

### TIMER1 database

TIMER1 (https://cistrome.shinyapps.io/timer/) database is a comprehensive analysis of the expression of NCAPD2 mRNA in various cancer tissues and normal tissues, which can explore the correlation between NCAPD2 expression and levels of immune invasion in different cancer types and generate a scatter plot. The “SCNA” module provided a comparison of the levels of tumor infiltration with different copy number alterations of NCAPD2 in LUAD and LUSC [18]. For the abbreviation of cancer names in the TCGA database, see Supplementary Table 1.

### GSCA database

GSCA database can detect the expression of NCAPD2 in different cancer stages (including stage I to stage IV). To explore the mRNA expression of NCAPD2 after DNA methylation in different cancers. Ultimately, we can explore the correlation between NCAPD2 and drug sensitivity [19].

### UALCAN database

UALCAN portal provides the total protein expression of NCAPD2 analysis option using data from Clinical Proteomic Tumor Analysis Consortium (CPTAC) [20]. Then enter the NCAPD2 gene and cancer on the website and click “Explore” module.

### GEPIA2 and Kaplan-Meier plotter

The GEPIA2 database (http://gepia2.cancer-pku.cn/#analysis) was used to analyze NCAPD2 mRNA expression levels in tumors without normal tissues. In addition, GEPIA2 can also detect the expression of NCAPD2 in OV and CESC, and the survival contribution of NCAPD2 in multiple cancer types and provides survival maps of Overall survival (OS) and Disease-free survival (DFS) [21]. The Kaplan Meier plotter is capable of assessing the correlation between the expression of NCAPD2 and survival in five tumor types including breast, ovarian, lung, gastric and liver cancer [22].

### GO enrichment analysis

NCAPD2 was analyzed using the STRING database (https://string-db.org/) which contains 20 highly significant proteins that interact with it [23]. Subsequently, We used GEPIA2 database to explore the top 60 similar genes of NCAPD2 in various cancers. Herein, we collected the interaction between two groups, and further identified their common members. After intersection analysis, a Venn diagram was formed. Next, we analyzed GO enrichment and Kyoto Encyclopedia of Genes and Genomes (KEGG) pathway analysis by using the database for annotation, visualization and integrated discovery (DAVID) database (https://david.ncifcrf.gov/) [24]. Then, we used TIMER1 to investigate the relationship of NCAPD2 with three interactors. After logging into the bioinformatics (http://www.bioinformatics.com.cn) website, we get visualization and modeling of biomedical data [25].

### cBioPortal database

The cBioPortal database (http://www.cbioportal.org/) provides the mutation frequency, copy number alteration and mutation type of NCAPD2. Herein, “TCGA pan-cancer atlas studies” was used to further detect NCAPD2 alterations in pan-cancer. Moreover, the correlation between mutation of NCAPD2 gene and progression-free survival (PFS) and DFS of patients can be explored in the “Survival” module [26].

### SangerBox database

We utilized the SangerBox website (http://www.sangerbox.com/tool) to evaluate the relationships between the NCAPD2 expression and immune cell types, microsatellite instability (MSI), tumor mutational burden (TMB), loss of heterozygosity (LOH), mutant-allele tumor heterogeneity (MATH), homologous recombination deficiency (HRD), ploidy and tumor stemness by the Pearson’s method [27]. We employed Estimation of Stromal and Immune cells in LUAD and LUSC using Expression data (ESTIMATE).

### TISIDB

We identified the relationship of NCAPD2 and two immunomodulators utilizing the portal of tumor immune system interaction (TISIDB) (http://cis.hku.hk/TISIDB/). Then, we explored the relevance of NCAPD2 expression and immune subtypes in LUAD and LUSC [28].

### HPA database

Analysis of NCAPD2 protein expression has been used the Human Protein Atlas (HPA) database in LUAD and LUSC (https://www.proteinatlas.org/).

### Statistical analysis

We plotted receiver operating characteristic (ROC) curves using the R package “survival ROC”, and the area under the ROC curve was used to assess the prognostic performance of the NCAPD2 score. RNA-sequencing expression profiles and corresponding clinical information for NCAPD2 were downloaded from the TCGA dataset (https://portal.gdc.com). R package was used to build Sankey diagram. CellMiner dataset on NCAPD2 and multiple drugs in different tumors were deteccted (https://discover.nci.nih.gov/cellminer/). Scatter graphs are built based on R software packages. *P* < 0.05 was considered statistically significant.

## Results

### NCAPD2 expression is up-regulated in pan-cancer

**Fig. 2:**
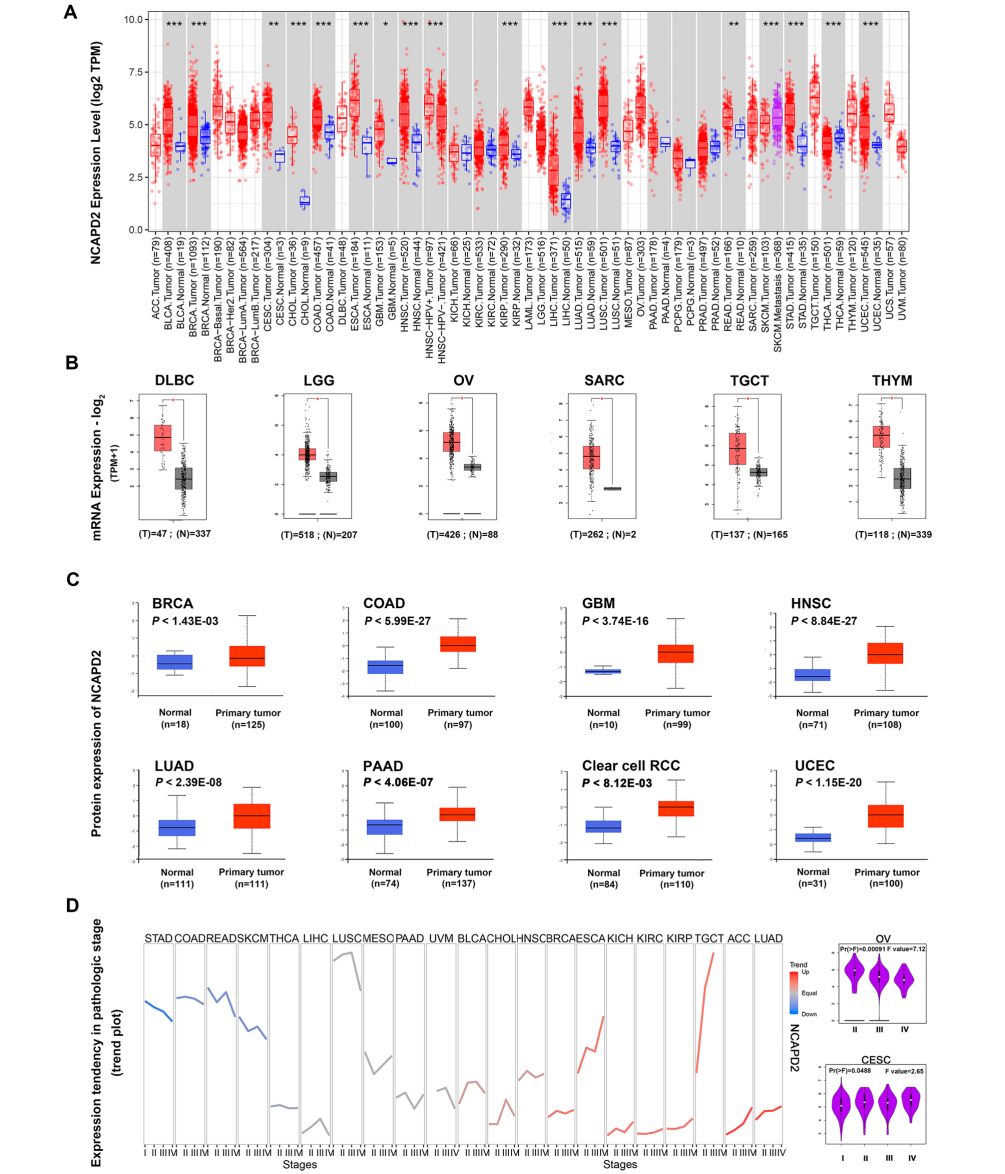
Expression of NCAPD2 in pan-cancer. (**A**) The NCAPD2 mRNA levels in the TIMER1 database. (**B**) The NCAPD2 mRNA expression in different cancers compared with normal tissues in GEPIA2 databases. (**C**) The protein expression of NCAPD2 in CTPAC. (**D**) the expression of NCAPD2 in different cancer stages (*P < 0.05, **P < 0.01, ***P < 0.001)

Compared with normal tissues, NCAPD2 mRNA expression was significantly increased in digestive, urinary, female reproductive and respiratory system tissues (Fig. 2A, all *P* < 0.05). Since TIMER1 does not currently include normal tissues, we analyzed the NCAPD2 mRNA expression of other tumor tissues in GEPIA2 database instead. Then, we discovered the mRNA expression of NCAPD2 was higher in various cancer types, including DLBC, LGG, OV, SARC, TGCT and THYM (Fig. 2B, all *P* < 0.05). However, the expression of NCAPD2 in ACC, LAML and UCS was no statistical significance (Supplementary Fig. 1). Based on UALCAN portal, the protein expression of NCAPD2 was also up-regulated among the BRCA, COAD, UCEC, Clear cell RCC, LUAC, PAAD, HNSC and GBM (Fig. 2C, all *P* < 0.05). Moreover, GSCA and GEPIA2 database showed that the expression of NCAPD2 was related to the stage of the certain cancers, and the expression level of NCAPD2 in LUAD, ACC, KIRP, BRCA, KIRC, ESCA and TGCT showed a gradual upward trend with the increase of clinical and pathological stage. In SKCM and LIHC, the expression of NCAPD2 decreased gradually with the increase of pathological stage (Fig. 2D, Supplementary Table 2, *P* < 0.05). These data suggested that NCAPD2 was highly expressed and played an oncogene role in multiple cancers.

### High NCAPD2 expression predicts poor prognosis in pan-cancer

**Fig. 3:**
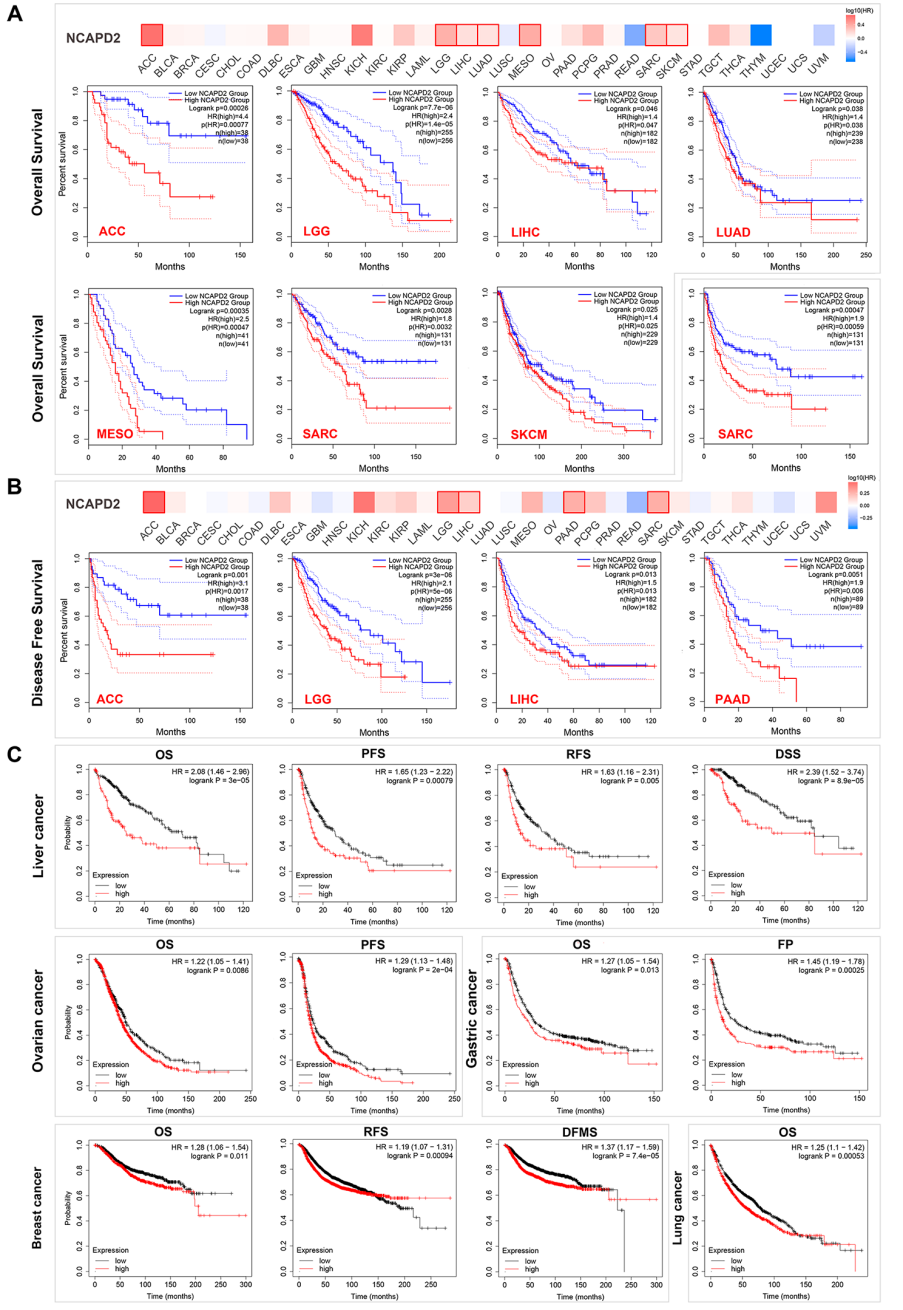
Survival analysis of NCAPD2 in pan-cancer. (**A**) The OS of patients with NCAPD2. (**B**) The DFS of patients with NCAPD2. (**C**) The survival of patients with NCAPD2 in breast, ovarian, gastric, lung and liver cancer (all *P* < 0.05)

In GEPIA2, we investigated that the OS of NCAPD2 high expression group was obviously shorter than the low one, including the ACC, LIHC, LGG, LUAD, MESO, SARC and SKCM (Fig. 3A, all *P* < 0.05). In addition, the DFS of NCAPD2 was significantly shorter in the high expression group than in the low expression group, covering ACC, LIHC, LGG, PAAD and SARC (Fig. 3B, all *P* < 0.05). Similarly, the expression of NCAPD2 was considerably associated with poor survival in breast cancer (OS: *P* = 0.011, RFS: *P* = 0.00094, DMFS: *P* = 7.4E-06), OV (OS: *P* = 0.0086, PFS: *P* = 2E-04), lung cancer (OS: *P* = 0.00053), gastric cancer (OS: *P* = 0.013, FP: *P* = 0.00025) and liver cancer (OS: *P* = 3E-05, RFS: *P* = 0.005, PFS: *P* = 0.00079, DSS: *P* = 8.9E-05) (Fig. 3C). These findings clearly showed that the high NCAPD2 expression was significantly associated with poor prognosis in cancer patients, which could be a important marker for all types of cancer.

### NCAPD2 and related genes are involved in immune processes in pan-cancer

**Fig. 4:**
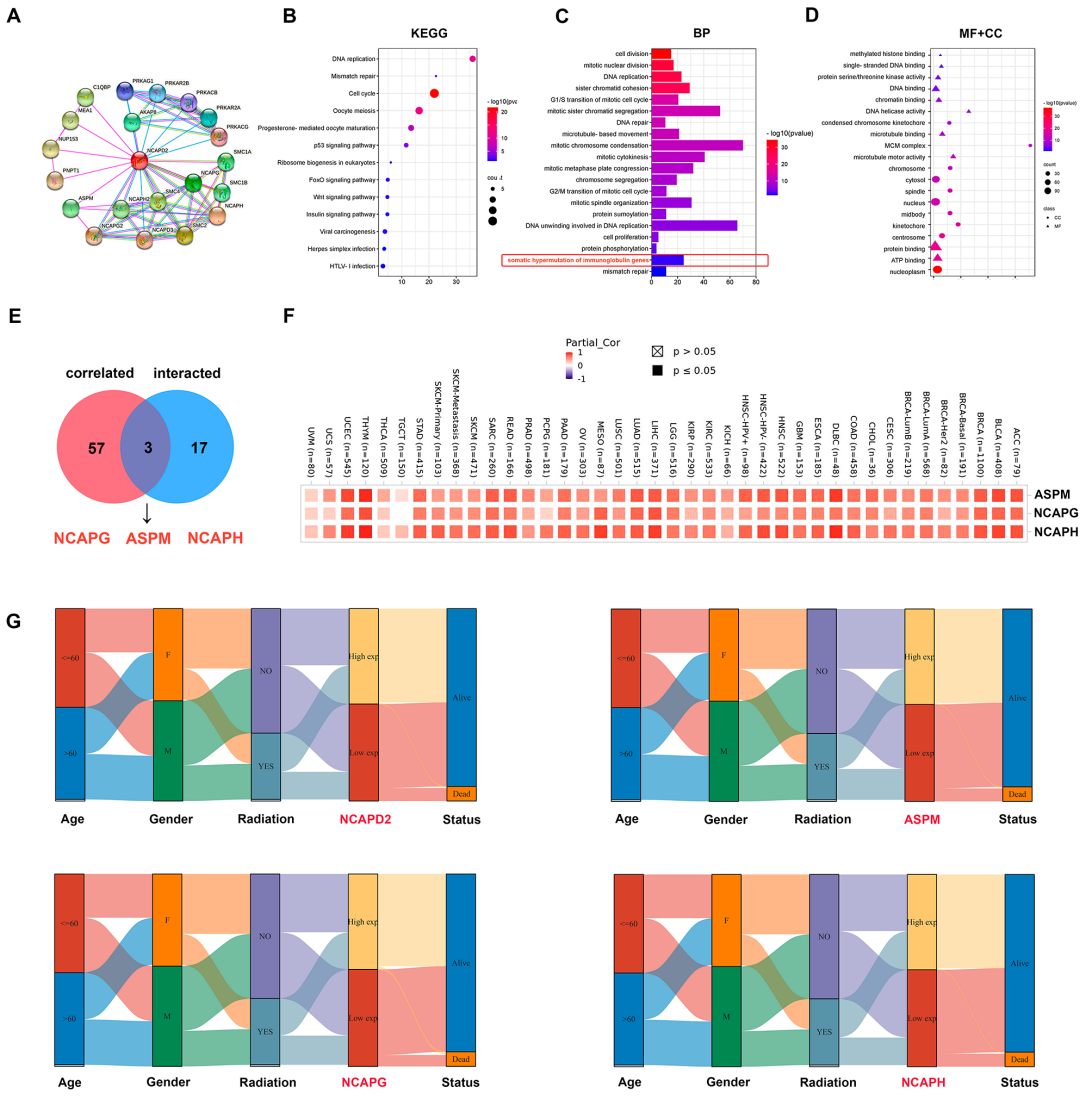
NCAPD2-related genes enrichment analysis. (**A**) A PPI network of 20 NCAPD2-interacted proteins. (**B**) The KEGG enrichment analysis of NCAPD2. (**C**) The BP enrichment analysis of NCAPD2. (**D**) The MF and CC enrichment analysis of NCAPD2. (**E**) Intersection analysis of NCAPD2 similar genes and NCAPD2-interacted proteins. (**F**) NCAPG, NCAPH and ASPM have strong positive correlations with NCAPD2 expression in different cancers. (**G**) Clinical expression levels of NCAPD2 and NCAPG, NCAPH, and ASPM in THYM

In order to evaluate the molecular mechanism of NCAPD2 in oncogenesis and progression, we then performed enrichment analysis. We analyzed a total of 20 NCAPD2-interacting proteins in the STRING database (Fig. 4A). In the following steps, the GEPIA2 was used to integrate the top 60 genes most similar to NCAPD2 (Supplementary Table 3). The KEGG analysis revealed that these 80 genes were primarily concentrated in ways related to the “cell cycle”, “oocyte meiosis”, “DNA replication” and “p53 signaling pathway” (Fig. 4B). The biological process (BP) analysis revealed that these genes were mainly related to “cell division”, “mitotic nuclear division”, “sister chromatid cohesion” and “hypermutation of immunoglobulin genes” (Fig. 4C). Interestingly, the somatic hypermutation of immunoglobulin genes participated in the immune response, suggesting that NCAPD2 may be related to the immune process. The findings of cellular component (CC) and molecular function (MF) were consistent with the above (Fig. 4D). In addition, the Venn diagram of the above-mentioned two groups obtained three common members, namely NCAPG, NCAPH and ASPM (Fig. 4E). NCAPG, NCAPH and ASPM have strong positive correlations with NCAPD2 expression in different cancers, especially in THYM (Fig. 4F). NCAPD2 was consistent with NCAPH, NCAPG and ASPM in the mulberry plot of data flow distribution in THYM classification with different clinicopathological parameters (Fig. 4G). Collectively, these evidences suggested that NCAPD2 might be involved in DNA repair, p53 signaling pathway, immune response and targeted ASPM protein.

### Alterations of NCAPD2 gene are associated with development and progression of pan-cancer

**Fig. 5:**
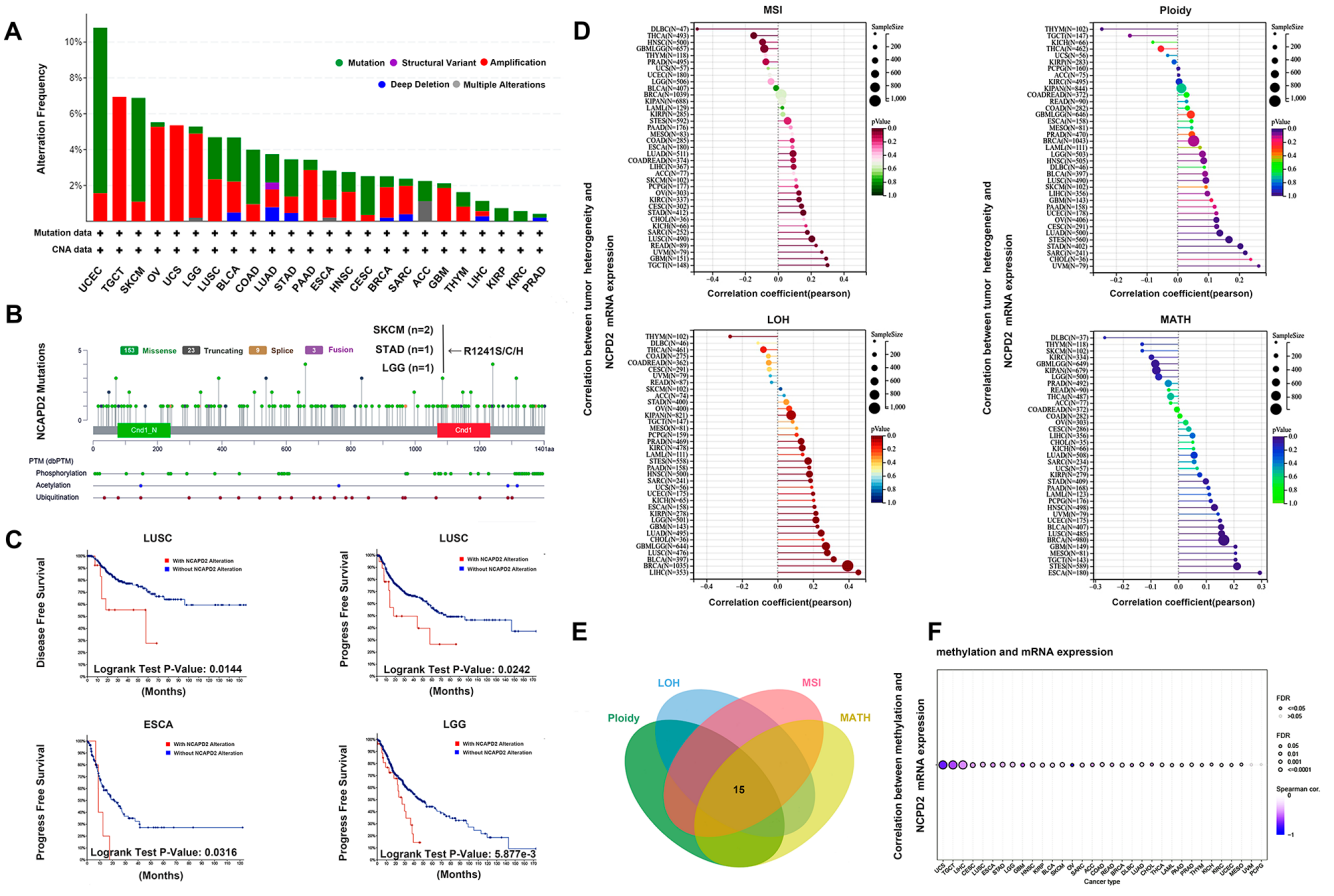
Alterations of NCAPD2 gene in pan-cancer. (**A**) The alteration frequency of NCAPD2 in pan-cancer. (**B**) The mutation types, sites and case number of NCAPD2 genetic alternation. (**C**) The survival of patients with NCAPD2 alternation. (**D**) Correlation between NCAPD2 expression level and ploidy, LOH, MSI, MATH. (**E**) Intersection analysis of NCAPD2 and ploidy, LOH, MSI, MATH. (**F**) The correlation between NCAPD2 mRNA expression level and methylation

It is widely acknowledged that genomic alteration is almost associated with tumorigenesis. In UCEC, SKCM and COAD, NCAPD2 alteration showed comparatively high mutation level, and the high amplification level of NCAPD2 in TGCT, UCS, OV (Fig. 5A). There were 153 missense, 23 truncating, 9 splice, and 3 fusion mutations between amino acids 0 and 1401. Among R1241S/C/H was the most frequent mutation site and discovered in STAD (n = 1), LGG (n = 1) and SKCM (n = 2) (Fig. 5B). Compared with patients with NCAPD2 alterations, the LUSC patients without altered NCAPD2 had better prognosis in PFS (*P* = 0.0242) and DFS (*P* = 0.0144). Both ESCA (*P* = 0.0316) and LGG (*P* = 5.877E-3) patients without NCAPD2 alterations had longer survival than with NCAPD2 alterations (Fig. 5C). Our findings indicated that NCAPD2 expression was significantly positively associated with MSI, LOH, MATH and ploidy in most tumors (Fig. 5D). Through the intersection analysis of the above four groups, we obtained 15 cancers in which NCAPD2 was changed in different genetic heterogeneity (Fig. 5E). Consistent with these observations, NCAPD2 DNA methylation was significantly negatively correlated with NCAPD2 gene expression in most cancers (Fig. 5F). The above results indicated that the gene alteration of NCAPD2 might promote the occurrence and development of tumors.

### NCAPD2 expression is associated with cancer stemness and drug sensitivity in pan-cancer

**Fig. 6:**
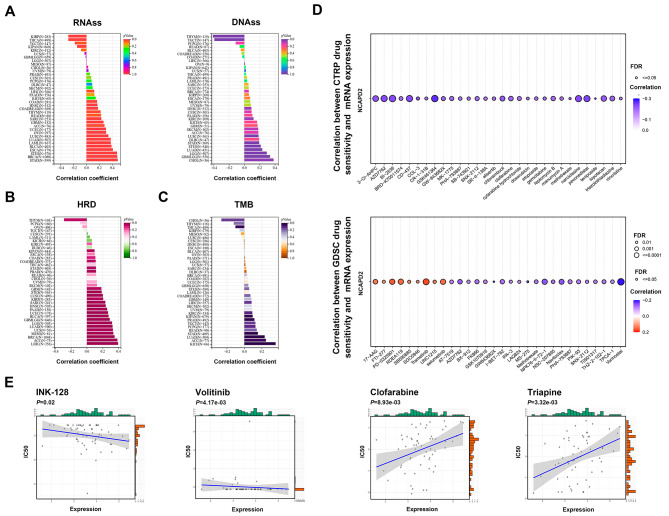
Relationship between NCAPD2 expression and cancer stemness, drug sensitivity. (**A**) Association between NCAPD2 and RNAss, DNAss. (**B**) Association between NCAPD2 and HRD. (**C**) Association between NCAPD2 and TMB. (**D**) Association between NCAPD2 and GDSC, CTRP drug sensitivity (Red indicated a positive correlation, with higher gene expression had greater susceptibility to the drug, while blue indicated the opposite). (**E**) Correlation between NCAPD2 and CellMiner drug sensitivity (*P* < 0.05)

Several studies have recently indicated that cancer stemness index plays a key role in tumor pathology and shows potential drug targets for anticancer therapies. We observed a positive correlation between NCAPD2 and cancer stemness: DNAss and RNAss (Fig. 6A). Consistently, NCAPD2 expression had a significantly positive correlation with HRD score in most cancers, which evaluated as predictors of response to neoadjuvant platinum-based therapy (Fig. 6B). Our studies showed that NCAPD2 expression had significant positive associations with TMB in most cancers (Fig. 6C). According to the data of GDSC and CTRP, the relationship between NCAPD2 expression level and drug sensitivity was analyzed. Our studies found that pevonedistat, topotecan, vorinostat, navitoclax and other drugs were highly sensitive to NCAPD2. In contrast, NCAPD2 was resistant to drugs such as trametinib and selumetinib (Fig. 6D). Using CellMiner, the expression of NCAPD2 in ICG-001 and Volitinib was negatively correlated with IC_50_, and positively with clofarabine and gemcitabine (Fig. 6E). We speculated that these indicators may benefit from predicting immunotherapies and NCAPD2 might be a specific molecular target for the study of targeted drugs.

### NCAPD2 is correlated with immune infiltration in LUAD and LUSC

**Fig. 7:**
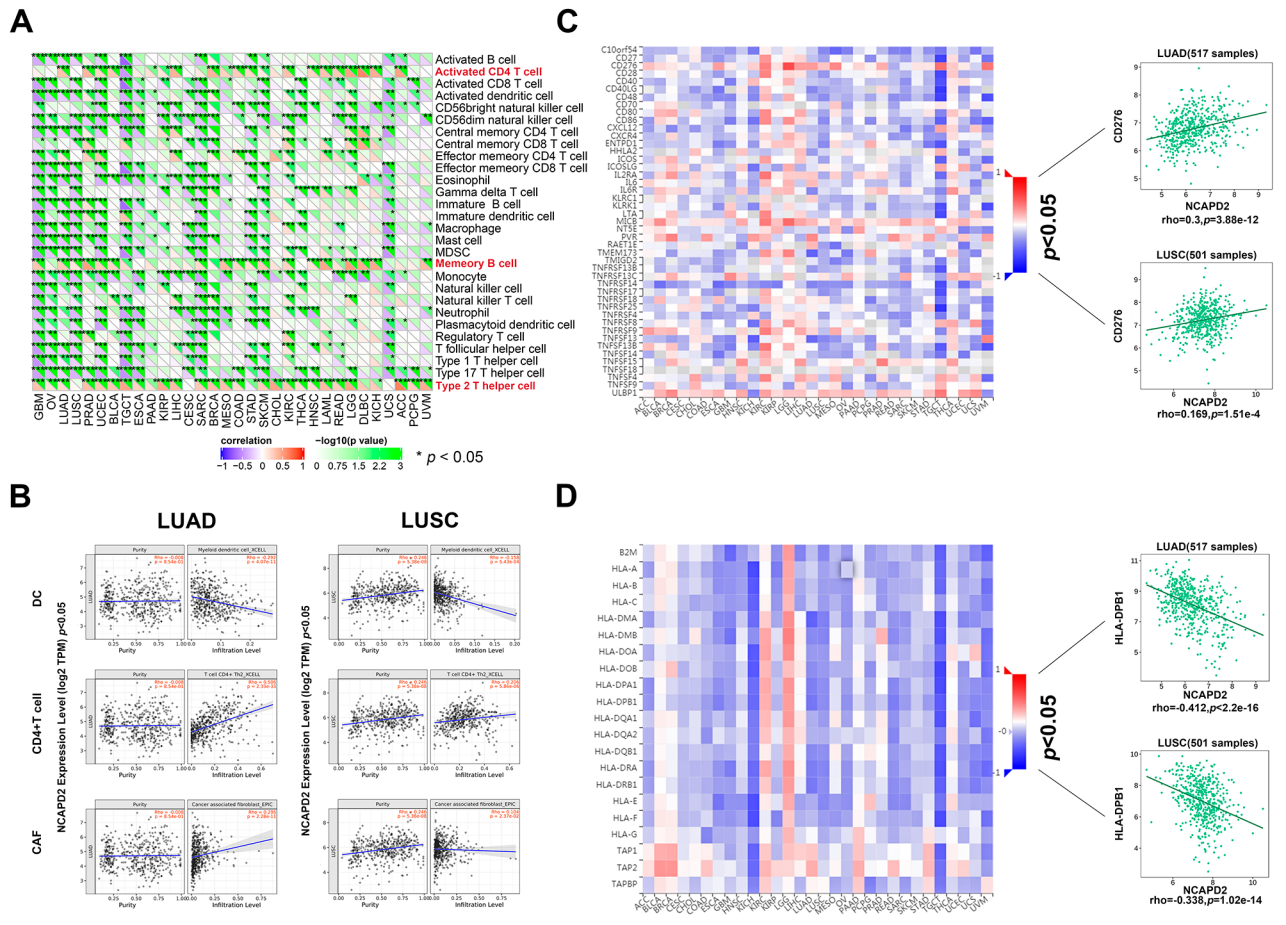
Relationship between NCAPD2 and immune infiltration in LUAD and LUSC. (**A**) Association between NCAPD2 and immune cell types. (**B**) Correlation between NCAPD2 and DCs, CD4^+^T cells, CAFs in LUAD and LUSC. (**C**) Correlation between NCAPD2 expression level and immunomodulators. (**D**) The relationship between NCAPD2 expression level and MHCs

On the basis of the above analysis, we speculated that NCAPD2 was related to different immune cell types in TME. The result showed that NCAPD2 was positively correlated with activated CD4^+^ T cells, Th2 and memory B cells in most tumors, such as LUAD, PRAD, etc. (r > 0, *P* < 0.05) (Fig. 7A). Since the pathologic types of the tumors were mostly squamous carcinoma and adenocarcinoma, we selected LUSC and LUAD as following studies. Further analysis showed that the relationship between NCAPD2 expression level and the infiltration of CAFs and CD4^+^T cells were positive in LUAD and LUSC, but negative in DCs (*P* < 0.05) (Fig. 7B). To further examine the association between NCAPD2 expression and immunomodulators, we identified that NCAPD2 expression was positive correlation linked with CD276 in LUAD and LUSC (r > 0, *P* < 0.05) (Fig. 7C). For major histocompatibility complex (MHC), there were negative correlations with NCAPD2 expression in HLA-DPB1 (r < 0, *P* < 0.05) (Fig. 7D). The above results confirmed that NCAPD2 may influence antitumor immunity by controlling TME composition, especially in LUAD and LUSC.

### NCAPD2 is a potential biomarker of immunotherapy in LUAD and LUSC

**Fig. 8:**
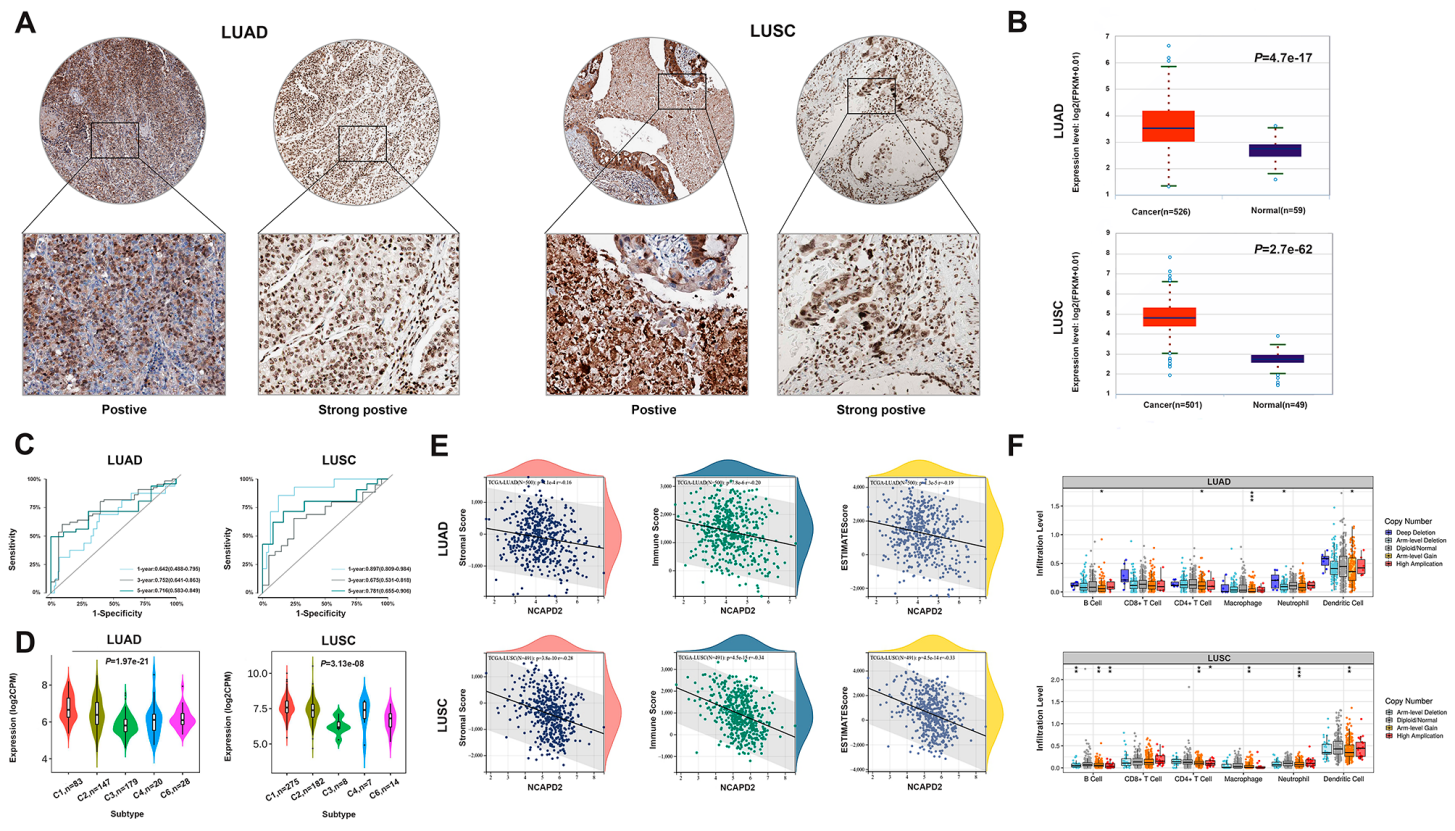
NCAPD2 was correlated with immunotherapy in LUAD and LUSC. (**A**) IHC staining of NCAPD2 in LUAD and LUSC tissues. (**B**) The expression of NCAPD2 in LUAD and LUSC. (**C**) NCAPD2 diagnostic value in LUAD and LUSC patients. (**D**) Associations between NCAPD2 expression and immune subtypes in LUAD and LUSC. (**E**) Associations between NCAPD2 expression and TME in LUAD and LUSC. (**F**) The distributions of TIICs at NCAPD2 SCNA category in LUAD and LUSC

IHC staining of HPA confirmed positive and strongly positive expression of NCAPD2 protein in LUAD and LUSC tissues (Fig. 8A). NCAPD2 gene expression was significantly higher in LUAD and LUSC (*P* < 0.05) (Fig. 8B). The diagnostic value was assessed using the ROC curve analysis in LUSC, the area under curve (AUC) was 0.897 at 1 year, 0.675 at 3 years and 0.781 at 5 years. Furthermore, the AUC in this risk signature was 0.642, 0.752 and 0.716 for predicting 1 year, 3 years and 5 years survival, respectively in LUAD (Fig. 8C). Our further study found that the expression level of NCAPD2 was closely correlated with immune subtypes in LUAD and LUCS (*P* < 0.05) (Fig. 8D). With the Pearson method, the NCAPD2 mRNA expression was negatively correlated with ESTIMATEScore, ImmuneScore and StromalScore in LUAD and LUSC (Fig. 8E, all *P* < 0.05). Compared with normal somatic copy number alterations (SCNA), the tumor infiltration levels were higher in DCs with different SCNA of NCAPD2 in LUAD and LUSC, and arm level gain frequently occurred in most TIICs (Fig. 8F, *P* < 0.05). These findings suggested that NCAPD2 could be an immune related biomarker on the efficacy of immunotherapy and a prognostic marker for LUAD and LUSC.

## Discussion

As a key cell cycle regulator, NCAPD2 was significantly increased and predicted poor prognosis in BRCA [29]. In our study, we found that the mRNA expression level of NCAPD2 was significantly increased in most cancers except ACC, LAML and UCS. One-point worth noting was that the expression level of NCAPD2 was positively correlated with clinical stage and shorter survival (OS and DFS) in most cancer patients. However, its expression in ACC is not significant, which might be related to the number of cases and needs further verification. Most notable, NCAPD2 had diagnostic value in patients with LUAD and LUSC. Therefore, our research provided insights into the application of NCAPD2 as pan-cancer prognostic markers.

As is well known, somatic mutation was one of the main mechanisms involved in tumorigenesis [30]. Hudnall et al. reported that NCAPD2 was amplified in all five cell lines of hodge lymphoma [31]. Studies have also revealed that a novel homozygous splice site variant in the NCAPD2 gene caused primary microcephaly [32]. Our studies showed that NCAPD2 has frequent alteration in pan-cancer, and amplification as the most commonly occurring type of alteration, whereas structural variant was the rarest. Subsequently, we found that NCAPD2 gene expression was related with the mark score of tumor heterogeneity, contains MSI, ploidy, MATH and LOH. Tumor heterogeneity is not only related to coding region mutations, but also included many epigenetic mechanisms, including DNA methylation and chromatin remodeling, that promote diversity within tumors [3]. Huang et al. confirmed that NCAPD2 was associated with two H3K9 demethylases, KDM3A and KDM4C [33]. Similarly, our studies indicated that NCAPD2 may regulate the tumor progression by mediating DNA repair and DNA methylation across cancers.

NCAPD2-interacted proteins and NCAPD2 similar genes in pan-cancer were integrated, and three common genes were obtained. Current researches showed that NCAPH up-regulated predicts a poor prognosis of prostate cancer patients and NCAPG promotes the progression of LUAD []. ASPM, an abnormal spindle like microcephaly associated protein, which is overexpressed in 27 cancers. Vincent et al. demonstrated that ASPM was an essential regulator of Wnt-Diseveled-3-β-catenin signaling and cancer stemness in prostate cancer, which can promote prostate cancer cells proliferation [35]. Another study identified that ASPM increased levels of immune cell infiltration in KIRC and LIHC [36]. Notably, enrichment analysis showed that NCAPD2 was associated with the Wnt signaling, which perhaps indicated NCAPD2 and ASPM may promote the development of various tumors immunity *via* Wnt signaling pathway.

In this study, another essential finding was that the expression of NCAPD2 gene is related to stemness index in most cancers. Higher DNAss and RNAss index values were associated with active BP in cancer stem cells and greater tumor dedifferentiation [37]. Importantly, the stemness index was significantly correlated with immune microenvironment content [38]. Our studies showed that NCAPD2 was significantly associated with immune infiltration in LUAD and LUSC. A recent study reported that the blockage of fibroblast growth factor/fibroblast growth factor receptor pathway can affect CAFs in LUAD, and inhibit the formation of lung epithelial cells [39]. In our research, CAFs are highly expressed in NCAPD2, which may be partly responsible for the poor prognosis in LUAD and LUSC patients. Kwak et al. revealed that the complement activation *via* a C3/C5-dependent pathway alters CD4 + T lymphocytes in lung cancer and drives immune escape mechanism [40]. Besides, the homeostasis formed by each subset of CD4 + T cells is of weight for maintaining the immune function of lung cancer patients [41]. In LUAD and LUSC, NCAPD2 expression was not only positively correlated with CD4 + T cells, but also Th2 cells, which may imply that the ratio of Th1 cells to Th2 cells in CD4 + T cells is unbalanced, thus promoting tumor growth.

Recent reports elucidated that B7-H3 (B7 homolog 3 protein, also known as CD276) controlled cancer progression and prolonged the survival of patients *via* targeted chimeric antigen receptor (CAR)-T cells and CAR-NK cells [42, 43]. Lyu et al. identified that HLA-DPB2/HLA-DPB1 axis exerts an anticancer role by recruiting TILs cells and NK cells into the TME, and combined with immune checkpoint inhibitors may optimize the immunotherapy for breast cancer [44]. Subsequently, the transcriptome analysis showed that stromal and immune components in TME is positively related to LUAD and LUSC, emphasized the impact of the interaction between tumor cells and immune cells. In particular, we discovered that NCAPD2 is higher expressed in the C1 and C2 immune subtype (immunotherapy sensitivity) of LUAD and LUSC. We speculate that targeting NCAPD2 may help predict efficacy of immunotherapy. Moreover, topotecan (a topoisomerase I inhibitor) was highly drug sensitive in NCAPD2-overexpression cancer cells in this study. Their studies were consistent with ours, topotecan has shown remarkable efficacy in the treatment of leukemia, retinoblastoma and neuroblastoma in clinical and trials study [45–47]. Interestingly, topotecan also was an commonly used chemotherapy drug for lung cancer which triggers DCs activation and cytokine production [48]. These findings revealed that topotecan combined with immunotherapy can effectively target NCAPD2 to against cancer progression, but the specific mechanism of action still needs to be further confirmed.

In conclusion, our comprehensive first evaluated the role of NCAPD2 expression in the pan-cancer. Further analysis illustrated the characterization of LUAD and LUSC and highlights its worthy study of immune response and immunotherapy. It is crucial to study the biological impact and the mechanism of NCAPD2 in vitro and in vivo. The results presented here may provide new perspectives for the investigation of NCAPD2 as a potential cancer target.

### Electronic supplementary material

Below is the link to the electronic supplementary material.

**Supplementary Material 1: Supplementary Figure 1**. Analysis of NCAPD2 expression levels in ACC, LAML and UCS based on GEPIA2 database

**Supplementary Material 2: Supplementary Table 1**. The cancer types included in the TCGA pan-cancer data

**Supplementary Material 3: Supplementary Table 2**. Correlation between the NCAPD2 expression and the clinical or pathologic stage (I-IV) in pan-cancer

**Supplementary Material 4: Supplementary Table 3**. The top 60 genes most similar to NCAPD2 in GEPIA2 database

## Data Availability

RNAseq data in TPM format for TCGA and GTEx for pan-cancer. Drug sensitivity data was provided by CellMiner (https://discover.nci.nih.gov/cellminer/home.do) and GSCA database (http://bioinfo.life.hust.edu.cn/GSCA/#/), which are publicly available. The data of TIMER1 database, GSCA database, UALCAN database, GEPIA2 database, Kaplan Meier plotter, cBioPortal database, SangerBox website, TISIDB database and HPA database were directly used for analysis.
