# Correction: NCAPD2 is a favorable predictor of prognostic and immunotherapeutic biomarker for multiple cancer types including lung cancer

**DOI:** 10.1186/s41021-024-00301-z

**Published:** 2024-02-13

**Authors:** Linyuan Feng, Yang Yang, Zhenhua Lin, Minghua Cui, Aihua Jin, Aili Cui

**Affiliations:** 1 Yanbian University Hospital, Yanji, China; 2 Key Laboratory of Pathobiology of High Frequency Oncology in Ethnic Minority Areas, Yanbian University, State Ethnic Affairs Commission, Yanji, China

**Correction to: Genes and Environment (2024) 46:2**

10.1186/s41021-023-00291-4

Following publication of the original article [1], the authors reported that “NCPAD2” need to be updated to “NCAPD2” in the article title.

The original article [1] has been corrected.
