# Supplementary material for: NCAPD2 is a favorable predictor of prognostic and immunotherapeutic biomarker for multiple cancer types including lung cancer
Source: Genes Environ. 2024 Jan 3;46:2. doi: 10.1186/s41021-023-00291-4 (PMC10763337; doi:10.1186/s41021-023-00291-4)

**Supplementary Figure 1.** Analysis of NCAPD2 expression levels in ACC, LAML and UCS based on GEPIA2 database.


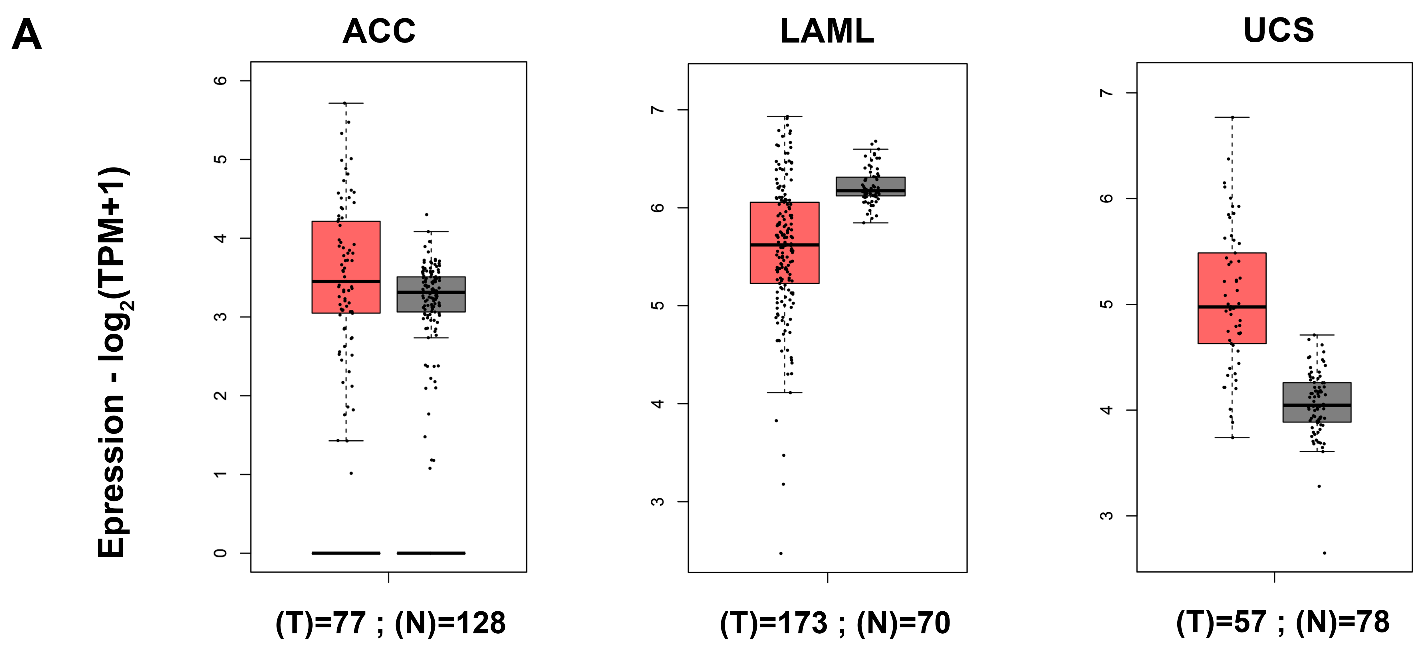

Supplement: Supplementary file 1 — Supplementary Material 1: Supplementary Figure 1. Analysis of NCAPD2 expression levels in ACC, LAML and UCS based on GEPIA2 database [file 41021_2023_291_MOESM1_ESM.docx]
