# Supplementary material for: NCAPD2 is a favorable predictor of prognostic and immunotherapeutic biomarker for multiple cancer types including lung cancer
Source: Genes Environ. 2024 Jan 3;46:2. doi: 10.1186/s41021-023-00291-4 (PMC10763337; doi:10.1186/s41021-023-00291-4)
Supplement: Supplementary file 2 — Supplementary Material 2: Supplementary Table 1. The cancer types included in the TCGA pan-cancer data [file 41021_2023_291_MOESM2_ESM.docx]

**Supplementary Table 1. Summary of the Pan-cancer Analysis of NCAPD2**

| **Cancer acronyms** | **Cancer full name** |
| --- | --- |
| ACC | Adrenocortical carcinoma |
| BRCA | Breast invasive carcinoma |
| CESC | Cervical squamous cell carcinoma and endocervical adenocarcinoma |
| COAD | Colon adenocarcinoma |
| DLBC | Lymphoid Neoplasm Diffuse Large B-cell Lymphoma |
| ESCA | Esophageal carcinoma |
| GBM | Glioblastoma multiforme |
| HNSC | Head and Neck squamous cell carcinoma |
| LAML | Acute Myeloid Leukemia |
| LGG | Brain Lower Grade Glioma |
| LIHC | Liver hepatocellular carcinoma |
| MESO | Mesothelioma |
| OV | Ovarian serous cystadenocarcinoma |
| PAAD | Pancreatic adenocarcinoma |
| SARC | Sarcomav |
| SKCM | Skin Cutaneous Melanoma |
| STAD | Stomach adenocarcinoma |
| TGCT | Testicular Germ Cell Tumors |
| THYM | Thymoma |
| UCEC | Uterine Corpus Endometrial Carcinoma |
| UCS | Uterine Carcinosarcoma |
| HCC | hepatocellular carcinoma |
| clear RCC | clear cell renal cell carcinoma |
