# Supplementary material for: NCAPD2 is a favorable predictor of prognostic and immunotherapeutic biomarker for multiple cancer types including lung cancer
Source: Genes Environ. 2024 Jan 3;46:2. doi: 10.1186/s41021-023-00291-4 (PMC10763337; doi:10.1186/s41021-023-00291-4)
Supplement: Supplementary file 3 — Supplementary Material 3: Supplementary Table 2. Correlation between the NCAPD2 expression and the clinical or pathologic stage (I-IV) in pan-cancer [file 41021_2023_291_MOESM3_ESM.docx]

**Supplementary Table 2. Correlation between the NCAPD2 expression and the clinical stage in pan-cancer.**

| **Cancer type** | **Stage type** | **Stage1** | **Stage2** | **Stage3** | **Stage4** | **Pval** | **FDR** |
| --- | --- | --- | --- | --- | --- | --- | --- |
|  |  | (Total sample size of cancer patients/clinical stage sample size) | | | |  |  |
| TGCT | clinical_stage | 4500/52 | 5200/17 | 7200/15 |  | 0.000878323 | 0.079829359 |
| KIRP | clinical_stage | 1000/140 | 1000/21 | 1200/29 | 1300/11 | 0.001906467 | 0.032377481 |
| CESC | clinical_stage | 3200/162 | 3600/69 | 3400/46 | 3800/22 | 0.014454507 | 0.334213041 |
| OV | clinical_stage |  | 4900/22 | 4100/246 | 3200/38 | 0.017218485 | 0.224087871 |
| UVM | clinical_stage |  | 1300/36 | 1400/40 |  | 0.182359592 | 0.67170062 |
| HNSC | clinical_stage | 3200/20 | 3400/98 | 3100/105 | 3600/285 | 0.259142698 | 0.585471017 |
| UCS | clinical_stage | 3300/22 | 3500/5 | 3500/20 | 4000/10 | 0.387362457 | 0.693665323 |
| ESCA | clinical_stage |  | 5700/30 | 4400/21 | 4700/11 | 0.523693406 | 0.876935599 |
| UCEC | clinical_stage | 2400/98 | 2400/24 | 2600/45 | 3100/10 | 0.534420785 | 0.886395793 |
| DLBC | clinical_stage | 3300/8 | 3500/17 | 3700/5 | 3300/12 | 0.736927376 | 0.99403642 |
| TGCT | pathologic_stage | 4100/56 | 6000/12 | 7500/14 |  | 0.000027594 | 0.012678238 |
| BRCA | pathologic_stage | 1800/182 | 2300/626 | 1800/252 | 1800/20 | 0.000067404 | 0.009125733 |
| KIRP | pathologic_stage | 1000/172 | 1100/22 | 1200/52 | 1500/15 | 0.000141804 | 0.00350199 |
| KIRC | pathologic_stage | 870/268 | 870/57 | 980/123 | 1300/84 | 0.000166598 | 0.001146767 |
| LUAD | pathologic_stage | 1700/277 | 2000/122 | 2200/84 | 2100/26 | 0.000842392 | 0.043322856 |
| ACC | pathologic_stage | 960/9 | 1200/37 | 1400/16 | 2000/15 | 0.002198045 | 0.095005734 |
| SKCM | pathologic_stage | 2900/78 | 2600/140 | 2600/171 | 2000/24 | 0.003916043 | 0.047471031 |
| LIHC | pathologic_stage | 690/173 | 870/87 | 1100/85 | 480/5 | 0.005832756 | 0.122199151 |
| ESCA | pathologic_stage | 3200/19 | 4600/78 | 3800/56 | 4700/9 | 0.044160913 | 0.271666421 |
| KICH | pathologic_stage | 820/21 | 1100/25 | 910/14 | 1600/6 | 0.05116422 | 0.363744832 |
| UVM | pathologic_stage |  | 1300/39 | 1400/36 |  | 0.242831821 | 0.982060966 |
| PAAD | pathologic_stage | 1200/21 | 1500/147 |  | 1300/5 | 0.278357116 | 0.504039585 |
| LUSC | pathologic_stage | 4000/245 | 4200/162 | 4100/84 | 3200/7 | 0.351864899 | 0.661759312 |
| STAD | pathologic_stage | 3000/57 | 2900/123 | 3000/171 | 2700/41 | 0.547249147 | 0.749599838 |
| BLCA | pathologic_stage |  | 2700/130 | 2900/140 | 2700/134 | 0.566601892 | 0.729564728 |
| COAD | pathologic_stage | 3000/45 | 3000/111 | 2900/81 | 2800/39 | 0.602097201 | 0.786180311 |
| READ | pathologic_stage | 3000/12 | 2800/26 | 2800/33 | 2800/13 | 0.617239759 | 0.877666166 |
| THCA | pathologic_stage | 1000/288 | 1000/51 | 1000/111 | 1000/57 | 0.628896036 | 0.729724321 |
| CHOL | pathologic_stage | 1400/19 | 1300/9 |  | 1700/7 | 0.713351256 | 0.999759909 |
| HNSC | pathologic_stage | 3000/27 | 3400/74 | 3200/81 | 3200/268 | 0.714005492 | 0.861095231 |
| MESO | pathologic_stage | 2200/10 | 1600/16 | 1900/45 | 1900/16 | 0.957308955 | 0.985171224 |
