# Supplementary material for: NCAPD2 is a favorable predictor of prognostic and immunotherapeutic biomarker for multiple cancer types including lung cancer
Source: Genes Environ. 2024 Jan 3;46:2. doi: 10.1186/s41021-023-00291-4 (PMC10763337; doi:10.1186/s41021-023-00291-4)
Supplement: Supplementary file 4 — Supplementary Material 4: Supplementary Table 3. The top 60 genes most similar to NCAPD2 in GEPIA2 database [file 41021_2023_291_MOESM4_ESM.docx]

**Supplementary Table 3. The top 60 genes most similar to NCAPD2 in GEPIA2 database.**

| **Gene Symbol** | **Gene ID** | **PCC** |
| --- | --- | --- |
| FOXM1 | ENSG00000111206.12 | 0.81 |
| RAD51AP1 | ENSG00000111247.14 | 0.8 |
| CDCA3 | ENSG00000111665.11 | 0.74 |
| KIF18B | ENSG00000186185.13 | 0.71 |
| KIF11 | ENSG00000138160.5 | 0.7 |
| NOP2 | ENSG00000111641.10 | 0.7 |
| RHNO1 | ENSG00000171792.10 | 0.69 |
| BUB1 | ENSG00000169679.14 | 0.69 |
| NCAPH | ENSG00000121152.9 | 0.69 |
| TPX2 | ENSG00000088325.15 | 0.68 |
| PLK1 | ENSG00000166851.14 | 0.68 |
| KIF23 | ENSG00000137807.13 | 0.68 |
| INCENP | ENSG00000149503.12 | 0.68 |
| KIF14 | ENSG00000118193.11 | 0.68 |
| KIF18A | ENSG00000121621.6 | 0.67 |
| GSG2 | ENSG00000177602.5 | 0.67 |
| ESPL1 | ENSG00000135476.11 | 0.67 |
| MKI67 | ENSG00000148773.12 | 0.67 |
| KIF2C | ENSG00000142945.12 | 0.66 |
| ZNF384 | ENSG00000126746.17 | 0.66 |
| KIF4A | ENSG00000090889.11 | 0.66 |
| CCNB2 | ENSG00000157456.7 | 0.66 |
| CCNA2 | ENSG00000145386.9 | 0.66 |
| CKAP2L | ENSG00000169607.12 | 0.66 |
| TMPO | ENSG00000120802.13 | 0.65 |
| TTK | ENSG00000112742.9 | 0.65 |
| MCM4 | ENSG00000104738.16 | 0.65 |
| NUSAP1 | ENSG00000137804.12 | 0.65 |
| RACGAP1 | ENSG00000161800.12 | 0.65 |
| GTSE1 | ENSG00000075218.18 | 0.65 |
| TICRR | ENSG00000140534.13 | 0.65 |
| HJURP | ENSG00000123485.11 | 0.65 |
| CCNF | ENSG00000162063.12 | 0.65 |
| MCM10 | ENSG00000065328.16 | 0.65 |
| LMNB2 | ENSG00000176619.10 | 0.65 |
| KIF20A | ENSG00000112984.11 | 0.64 |
| ARHGAP11A | ENSG00000198826.10 | 0.64 |
| LMNB1 | ENSG00000113368.11 | 0.64 |
| ASPM | ENSG00000066279.16 | 0.64 |
| CLSPN | ENSG00000092853.13 | 0.64 |
| CENPO | ENSG00000138092.10 | 0.64 |
| DLGAP5 | ENSG00000126787.12 | 0.64 |
| CENPF | ENSG00000117724.12 | 0.64 |
| CENPI | ENSG00000102384.13 | 0.63 |
| CCDC77 | ENSG00000120647.9 | 0.63 |
| STIL | ENSG00000123473.15 | 0.63 |
| KIF20B | ENSG00000138182.14 | 0.63 |
| SGOL1 | ENSG00000129810.14 | 0.63 |
| MCM2 | ENSG00000073111.13 | 0.62 |
| MCM8 | ENSG00000125885.13 | 0.62 |
| NCAPG | ENSG00000109805.9 | 0.62 |
| POLQ | ENSG00000051341.13 | 0.62 |
| SGOL2 | ENSG00000163535.17 | 0.62 |
| ORC1 | ENSG00000085840.12 | 0.62 |
| CEP55 | ENSG00000138180.15 | 0.62 |
| GINS1 | ENSG00000101003.9 | 0.62 |
| KIAA1524 | ENSG00000163507.13 | 0.62 |
| CDCA5 | ENSG00000146670.9 | 0.62 |
| SENP1 | ENSG00000079387.13 | 0.61 |
| HMMR | ENSG00000072571.19 | 0.61 |
